# Unlocking the Growth Potential of Poplar: A Novel Transcriptomic-Metabolomic Approach to Evaluating the Impact of Divergent Pruning Strategies

**DOI:** 10.3390/plants13233391

**Published:** 2024-12-03

**Authors:** Xiaoting Liu, Kewei Cai, Qinhui Zhang, Weizi An, Guanzheng Qu, Luping Jiang, Fusen Wang, Xiyang Zhao

**Affiliations:** 1 National Key Laboratory of Forest Genetics and Breeding, Northeast Forestry University, Harbin 150040, China; 2021032132@nefu.edu.cn (X.L.); gzqu@nefu.edu.cn (G.Q.); 2 Jilin Provincial Key Laboratory of Tree and Grass Genetics and Breeding, College of Forestry and Grassland Science, Jilin Agricultural University, Changchun 130118, China; ckw1006@nefu.edu.cn (K.C.); qhzhang@nefu.edu.cn (Q.Z.); awz1936815850@outlook.com (W.A.); jiangluping@jlau.edu.cn (L.J.); 3 Qiqihar Branch of Heilongjiang Academy of Forestry, Qiqihar 161000, China; wangfs66@163.com

**Keywords:** pruning, transcriptome, metabolome, starch and sucrose biosynthesis, plant hormone signal transduction

## Abstract

Pruning is a common forest-tending method; its purpose is to promote growth and improve the overall stand quality. Poplar is a fast-growing, broad-leaved tree species with high ecological and economic value. It is a common management method to promote its growth by pruning and adjusting the spatial structure of the stand, but its potential regulatory mechanism remains unclear. In this study, transcriptome and metabolome data of different parts at all pruning intensities were determined and analyzed. The results showed that 7316 differentially expressed genes were identified in this study. In the plant hormone signal transduction pathway, candidate genes were found in eight kinds of plant hormones, among which the main expression was gibberellin, auxin, and brassinosteroid. Some candidate gene structures (beta-glucosidase, endoglucanase, hexokinase, glucan endo-1, 3-beta-D-glucosidase, beta-fructofuranosidase, fructokinase, maltase-glucoamylase, phosphoglucomutase, and sucrose) were specifically associated with starch and sucrose biosynthesis. In the starch and sucrose biosynthesis pathway, D-fructose 6-phosphate, D-glucose 1,6-bisphosphate, and glucose-1-phosphate were the highest in stems and higher in the first round of pruning than in no pruning. The bHLH plays a key role in the starch and sucrose synthetic pathway, and AP2/ERF-ERF is important in the plant hormone signal transduction pathway. These results laid a foundation for understanding the molecular mechanism of starch and sucrose biosynthesis and provided a theoretical basis for promoting tree growth through pruning.

## 1. Introduction

Pruning is a common forest-tending method for artificially removing some living, all dying, and dead branches in the lower crown that do not contribute significantly to growth. Its purpose is to promote growth, cultivate straight dry type, improve the health status of the forest stand, and comprehensively improve the quality of the stand. It is widely used to manage *Fokienia hodginsii* (Dunn) Henry et Thomas [1], *Pinus koraiensis* Sieb. et Zucc. [2], and other artificial forests. This is an important method to produce non-jointed trees and landscape tree species with high economic value. Relevant studies have shown that moderate pruning using this technology can improve the ventilation and light transmission rates among forests [3], reduce water and nutrient consumption by dead branches, and promote the circulation and full utilization of water and nutrients in trees, which is conducive to forest regeneration and growth. To a certain extent, pruning can alleviate stand degradation and improve stand productivity [4]. It significantly changes the distribution of biomass and sharpness of the trunk, reduces the formation of knots [1], and is conducive to the formation of high-quality wood.

Poplar is a general term for *Populus* plants of *Salicaceae*. It is mainly distributed in the subtropical, temperate, and cold temperate regions of the Northern Hemisphere and has over 100 species [5]. It has the characteristics of a straight and round trunk, developed roots, a high survival rate of afforestation, fast growth, strong adaptability, and a short rotation period. It is one of the three most important fast-growing tree species for the intensive management of industrial materials in short rotation periods worldwide [6]. Poplar wood, with its lightweight and white texture, is also an important raw material for pulp and artificial board [7]. In China, poplar is mainly used in industry, civil materials, soil and water conservation, landscaping, and the construction of shelterbelts. It is an important component of China’s three northern shelterbelts and fast-growing high-yield forests; thus, it has high ecological and economic value.

Previous studies on poplar have mainly focused on cultivation management [8], variety breeding [9], pest control [10], fingerprint construction [11], molecular breeding [12], soil element content determination in forests, and determination of physical and chemical properties [13,14]. In this study, a combined transcriptome and metabolome analysis of roots, stems, and leaves under different pruning treatments was conducted to clarify the differences in the content of primary metabolites. The key regulatory gene expression was analyzed under different treatments to provide a theoretical basis for the effects of different pruning treatments on stand yield.

## 2. Results

### 2.1. Measurement of Plant DBH Growth

**Figure 1:**
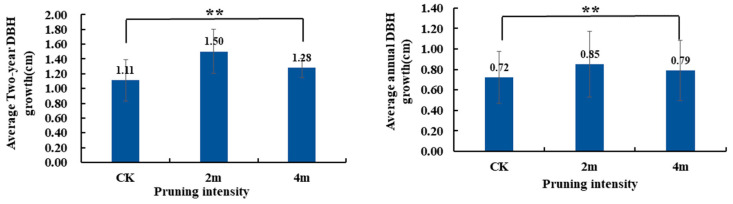
The DBH growth of poplar under different pruning treatments. ** significant at the 0.01 level.

Compared to CK, the average annual DBH growth and the average 2-year DBH growth after pruning treatment was improved, and the annual DBH growth under different pruning treatments differed significantly (*p* < 0.01; Figure 1). Among them, 2m had the best pruning effect.

### 2.2. Identification and Enrichment Analysis of Differentially Accumulated Metabolites (DEMs)

**Figure 2:**
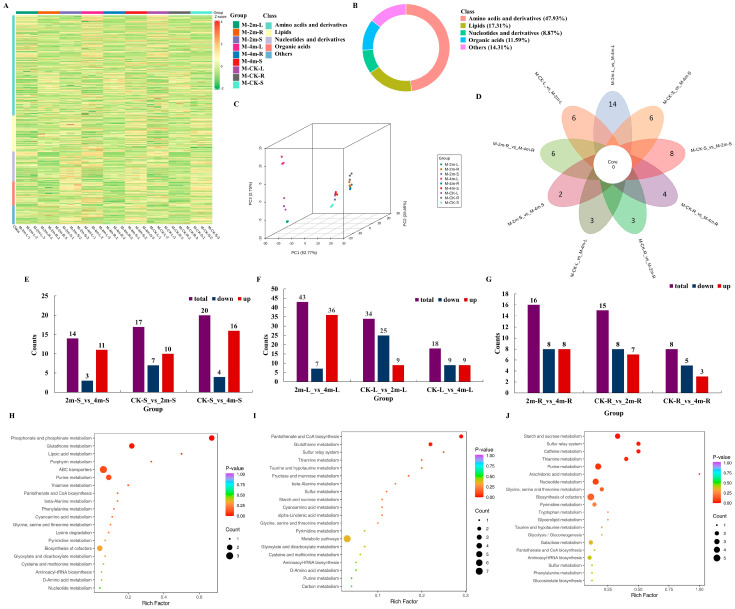
Identification and enrichment analysis of differentially accumulated metabolites. (**A**) Cluster heat map of all differentially accumulated metabolites in all group. (**B**) The classification of total metabolites in root, stem, and leaf samples. (**C**) PCA of the metabolome data from all groups. (**D**) Venn diagram of DEMs for all the comparative groups. (**E**) Statistical analysis of upregulated and downregulated DEMs for the comparison group of all stems. (**F**) Statistical analysis of upregulated and downregulated DEMs for the comparison group of all leaves. (**G**) Statistical analysis of upregulated and downregulated DEMs for the comparison group of all roots. (**H**) KEGG enrichment analysis of DEMs for the comparison group of all roots. (**I**) KEGG enrichment analysis of DEMs for the comparison group of all stems. (**J**) KEGG enrichment analysis of DEMs for the comparison group of all leaves.

The study revealed variations in metabolite composition and quantity between CK and pruning. Using both positive and negative ion modes, LC-MS/MS was employed for an extensive metabolomics analysis to investigate the differences in metabolite accumulation and their corresponding biological functions between CK and pruning. We identified 699 metabolic products, mainly consisting of five types of metabolites: amino acids and derivatives (335, 47.93%), lipids (121, 17.31%), nucleotides and derivatives (62, 8.87%), organic acids (81, 11.59%), and other metabolites (100, 14.31%) (Figure 2A,B). We found 79 saccharides and three key differentiating metabolites in the ‘starch and sucrose metabolism’ pathway in the study. Hierarchical clustering analysis was performed on the accumulation patterns of metabolites among the different samples under different treatments. Cluster analysis showed that all samples were divided into three groups, consistent with the PCA results (Figure 2C). DEMs from all paired groups in different parts were further identified using a VIP value ≥ 1 and fold change ≥ 2 or ≤ 0.5. Venn diagram (Figure 2D) and histogram show the results. A total of 43 (36 upregulated and seven downregulated), 34 (nine upregulated and 25 downregulated), 18 (nine upregulated and nine downregulated), 16 (eight upregulated and eight downregulated), 15 (seven upregulated and eight downregulated), eight (three upregulated and five downregulated), 14 (11 upregulated and three downregulated), 17 (10 upregulated and seven downregulated), and 20 (16 upregulated and four downregulated) metabolites were significantly different in 2m-L versus 4m-L, CK-L versus 2m-L, CK-L versus 4m-L, 2m-R versus 4m-R, CK-R versus 2m-R, CK-R versus 4m-R, 2m-S versus 4m-S, CK-S versus 2m-S, and CK-S versus 4m-S, respectively (Figure 2E–G).

The DEMs in the CK-R versus 2m-R versus 4m-R groups were divided into 24 KEGG pathways, while the number of enriched metabolic pathways in the CK-S versus 2m-S versus 4m-S and CK-L versus 2m-L versus 4m-L groups were 23 and 41, respectively. Among the top 20 enrichment pathways, ‘glutathione metabolism’, ‘pantothenate and CoA biosynthesis metabolism’, ‘metabolic pathways’, ‘starch and sucrose metabolism’, and ‘sulfur metabolism’ were the most significantly enriched pathways for the CK-R versus 2m-R versus 4m-R (Figure 2H), CK-S versus 2m-S versus 4m-S (Figure 2I), and CK-L versus 2m-L versus 4m-L (Figure 2J) groups. According to the statistical results of KEGG analysis, we found that the ‘starch and sucrose metabolism’ pathway was found in the CK-R versus 2m-R versus 4m-R, CK-S versus 2m-S versus 4m-S, and CK-L versus 2m-L versus 4m-L in most paired groups. Starch and sucrose metabolism was found in most paired groups, consistent with the enrichment analysis results of DEGs.

### 2.3. Identification and Enrichment Analysis of DEGs

In this study, 27 transcript libraries were constructed from three different treatments across three plant parts, followed by paired-end sequencing using an Illumina HiSeq 2500 platform. The raw reads ranged from 43,944,654 to 64,601,908. After filtering low-quality and adapter sequences, the clean reads ranged from 40,280,562 to 63,341,476. Specifically, the size of clean reads varied from 6.04 to 9.5 Gb, with an average of 6.99 Gb. The average contents of Q20 and Q30 were 97.01% and 91.85%, respectively, whereas the GC content ranged from 43.26% to 44.22%, with an average of 43.70% (Table S1).

**Figure 3:**
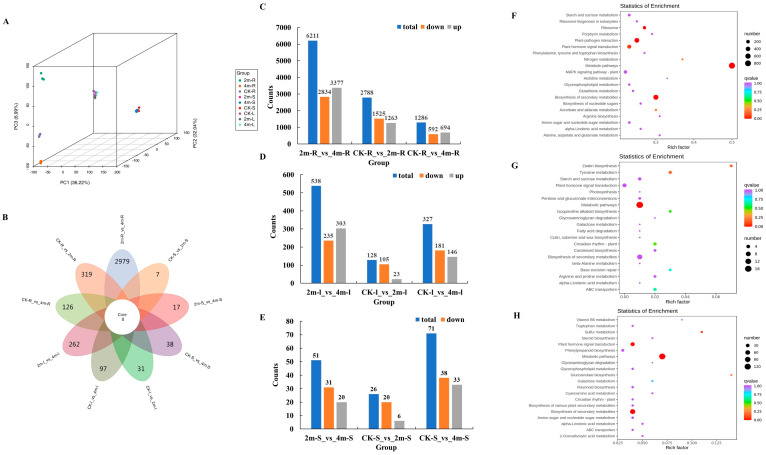
Identification and enrichment analysis of DEGs for all comparison groups. (**A**) PCA of the transcriptome data from all the groups. (**B**) Venn diagram of DEGs for all the comparative groups. (**C**) Statistical analysis of upregulated and downregulated DEGs for the comparison group of all roots. (**D**) Statistical analysis of upregulated and downregulated DEGs for the comparison group of all leaves. (**E**) Statistical analysis of upregulated and downregulated DEGs for the comparison group of all stems. (**F**) KEGG enrichment analysis of DEGs for the comparison group of all roots. (**G**) KEGG enrichment analysis of DEGs for the comparison group of all stems. (**H**) KEGG enrichment analysis of DEGs for the comparison group of all leaves.

Principal component analysis (PCA) was performed using the FPKM value. The results displayed that the samples of the three parts were divided into different units, with PC1, PC2, and PC3 accounting for 36.22%, 32.04%, and 6.99%, respectively (Figure 3A).

In this study, 7316 DEGs were found (Figure 3B), with 6211 (3377 upregulated and 2834 downregulated) in 2m-R versus 4m-R groups having the most in the root, followed by 2788 (1263 upregulated and 1525 downregulated) in the CK-R versus 2m-R, and 1286 (694 upregulated and 592 downregulated) in the CK-R versus 4m-R group (Figure 3C). The CK-S versus 4m-S groups had the highest DEGs in the stem, with 71 (33 upregulated and 38 downregulated), followed by 2m-S versus 4m-S with 51 (20 upregulated and 31 downregulated), and 26 (six upregulated and 20 downregulated) DEGs were identified in the CK-S versus 2m-S group (Figure 3E). The 2m-L versus 4m-L groups had the highest in stem, with 538 (303 upregulated, 235 downregulated), followed by CK-L versus 4m-L with 327 (146 upregulated and 181 downregulated), and 128 DEGs were identified in the CK-L versus 2m-L group (23 upregulated and 105 downregulated) (Figure 3D).

KEGG pathway analysis was performed to identify the biological functions of the DEGs obtained from the comparative transcriptome analysis. Next, KEGG enrichment analysis was performed to identify the key metabolic pathways of the DEGs in the roots, stems, and leaves. The DEGs in the CK-R versus 2m-R versus 4m-R groups were divided into 134 KEGG pathways, while the number of enriched metabolic pathways in the CK-S versus 2m-S versus 4m-S and CK-L versus 2m-L versus 4m-L groups were 30 and 86, respectively. Among the top 20 enrichment pathways, ‘metabolic pathways’, ‘biosynthesis of secondary metabolites’, ‘plant hormone signal transduction’, ‘plant-pathogen interaction’, and ‘starch and sucrose metabolism’ were the most significantly enriched (corrected *p*-value < 0.05) pathways for CK-R versus 2m-R versus 4m-R (Figure 3F), and CK-S versus 2m-S versus 4m-S (Figure 3G), and CK-L versus 2m-L versus 4m-L (Figure 3H) groups. According to the statistical results of KEGG analysis, we found that the ‘plant hormone signal transduction’ and ‘starch and sucrose metabolism’ pathways were found in the CK-R versus 2m-R versus 4m-R, CK-S versus 2m-S versus 4m-S, and CK-L versus 2m-L versus 4m-L groups.

### 2.4. Expression Analysis of Genes Involved in Plant Hormone Signal Transduction

**Figure 4:**
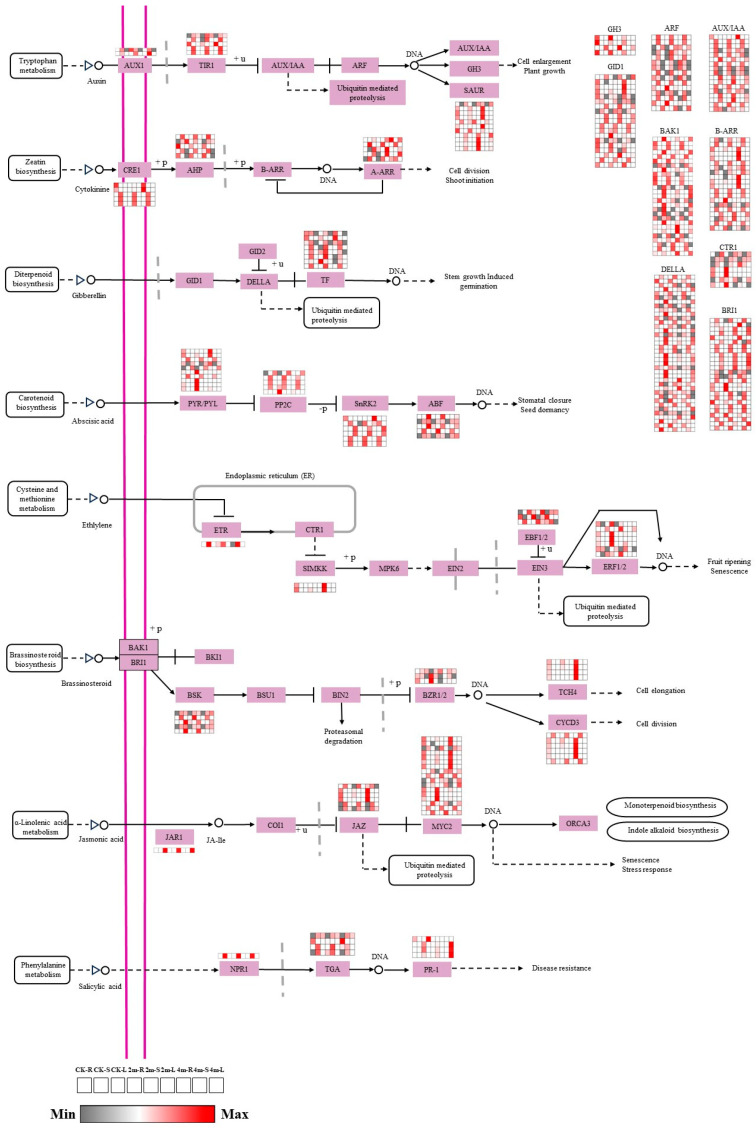
Expression analysis of DEGs involved in phytohormone biosynthesis and signal transduction. GID1, GA receptor GID1; TF, phytochrome-interacting factor 4; DELLA, DELLA protein; GID2, GA receptor GID2; AUX1, IAA influx carrier 1; TIR1, transport inhibitor response 1; AUX/IAA, IAA-responsive protein IAA; ARF, IAA response factor; GH3, gretchen hagen 3; SAUR, small IAA upregulated RNA; BAK1, BR insensitive 1-associated receptor kinase 1; BRI1, protein BR insensitive 1; BSK, BR-signaling kinase; BSU1, serine/threonine-protein phosphatase; BIN2, BR insensitive 2; BZR1/2, BR resistant 1/2; TCH4, xyloglucan: xyloglucosyl transferase; CYCD3, cyclin D3.

Given the specific accumulation patterns of phytohormones during plant development, a more in-depth investigation was conducted into the key genes implicated in the biosynthetic and signal transduction pathways of three crucial phytohormones. The expression profiles of these genes were subsequently analyzed through the use of a heat map. Initially, we identified 65, 54, and 69 candidate structural genes associated with gibberellin (GA), auxin, and brassinosteroid (BR) biosynthesis and signal transduction pathways, respectively, using gene annotation from different public databases (Figure 4). GA can be sensed and bound to the GID1 receptor. When GA enters the C-terminal pocket structure of GID1 at a high concentration, the GID1 protein undergoes conformational changes, and the N-terminal extension structure covers the pocket structure and forms a hydrophobic surface. This hydrophobic surface promotes the formation of GID1/GA/DELLA complex and releases DELLA inhibition of key downstream regulators, thus regulating various biological processes. The GA-GID1-DELLA signal transduction pathway contains 19 genes encoding GID1, 38 genes encoding DELLA, and eight genes encoding transcription factors (TFs). Most TF genes showed relatively high expression levels in all stems; among them, 2m-S had the highest expression level under different pruning intensities. However, two TF protein-encoding genes (*POPTR_011G129500v3* and *POPTR_001G410600v3*) were downregulated in all stems.

Auxin signal transduction involves three protein components: auxin receptor-related SCF complex (SKP1, Cullin, and F-box complex), auxin protein (Aux/IAA), and auxin response factor (ARF). Early response genes included Aux/IAA, GH1, GH3, GH2/4, and the SAUR gene family. There are four main auxin hormone signaling pathways: TIR1/AFB Aux/IAA/TPL-ARFs, TMK1-IAA32/34-ARFS, TMK1/ABP1-ROP2/6-PINs or RICs, and SKP2AE2FC/DPB. Two genes encode AUXI, five genes encode TIR1, 17 genes encode AUX/IAA, 15 genes encode ARF, and 11 genes encode SAUR. Most were highly expressed in stems of different pruning intensities; four genes encoded GH3, and most were highly expressed in roots of different pruning intensities.

BR binds to the extracellular domain of BRI1 to activate its intracellular kinase domain, and activated BRI1 can phosphorylate its negative regulatory factor BKI1 and dissociate it from the plasma membrane so that BRI1 can bind to its co-receptor BAK1. BRI1 and BAK1 fully activate BR signaling via sequential phosphorylation. Subsequently, BRI1 phosphorylates BSK1 and CDG1 and activates them. BSKs and CDG1 phosphorylated and activated BSU1, and the activated BSU1 dephosphorylated and inactivated BIN2, thereby removing the inhibitory function of BIN2 on BES1/BZR1. Finally, the transmission of the BR signal enables a group of six members of the TF family, BES1/BZR1 family, to accumulate in a non-phosphorylated state and activate downstream transcriptional regulation in the nucleus, thereby regulating plant growth and development and its response to environmental stimuli. In this study, four TCH4-encoding genes were highly expressed in all roots, with 4m-R expression being the highest under-pruning intensity in all root parts. Additionally, genes encoding BR-insensitive 1-associated receptor kinase 1 (BAK1), protein BR insensitive 1 (BRI1), BR-signaling kinase (BSK), BR resistant 1/2 (BZR1/2), and cyclin D3 (CYCD3) exhibited varied expression levels in response to different pruning intensities across all plant parts. These findings suggest significant gene variations related to the IAA, GAA, BR biosynthesis, and signal transduction pathways.

### 2.5. Identification and Expression Analysis of DEGs and DEMs in the Starch and Sucrose Metabolism Pathway

**Figure 5:**
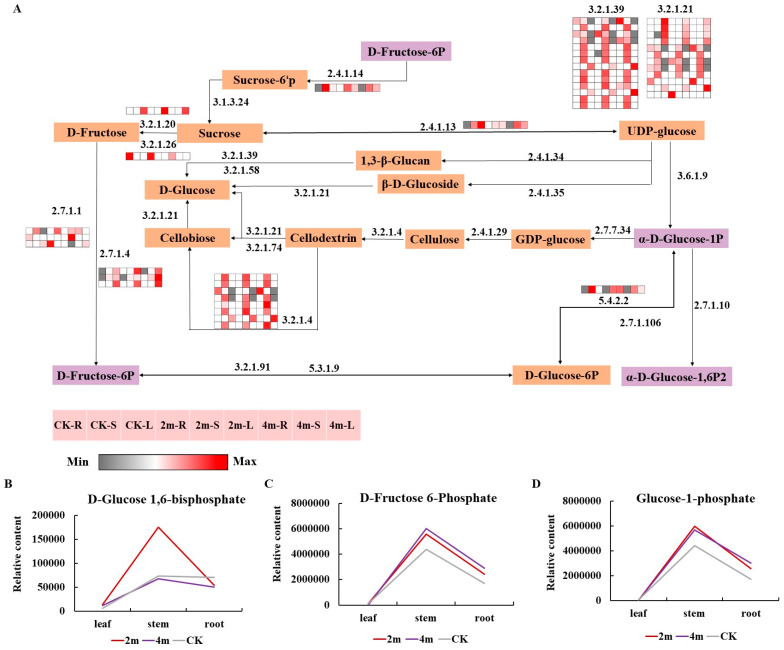
(**A**) Common genes involved in starch and sucrose metabolism were significantly expressed under different pruning intensities. 3.2.1.21, beta-glucosidase; 3.2.1.39, glucan endo-1,3-beta-D-glucosidase; 2.7.1.1, hexokinase; 2.7.1.4, fructokinase; 3.2.1.4, endoglucanase; 3.2.1.20, maltase-glucoamylase; 2.4.1.13, sucrose synthase; 2.4.1.14, sucrose-phosphate synthase; 5.4.2.2, phosphoglucomutase; 3.2.1.26, beta-fructofuranosidase. Relative contents of (**B**) D-glucose 1,6-bisphosphate, (**C**) D-fructose 6-phosphate, and (**D**) glucose-1-phosphate.

Starch and sucrose are important for plant photosynthesis, providing usable energy and are the basis for plant growth. KEGG enrichment analysis of DEGs and DEMs revealed that starch and sucrose metabolism pathways were common to all plant parts under different pruning intensities (Figure 5). Therefore, we evaluated the expression patterns of key genes and metabolites related to the starch and sucrose metabolism pathways. In this study, three DEMs (D-fructose 6-phosphate, D-glucose 1,6-bisphosphate, and glucose-1-phosphate) and 45 DEGs were identified and showed differential expression under pruning intensity in all parts. The relative contents of the three different metabolites in different parts were in the order of stem > root > leaf. In stems, the relative content of three metabolites under 2m pruning treatment was higher than CK, whereas D-glucose 1,6-bisphosphate and glucose-1-phosphate were the highest under 2m pruning treatment. In the 2m and 4m treatment groups, the relative content of D-glucose 1,6-bisphosphate was higher in leaves than in CK but lower in roots than in CK. The relative contents of D-fructose 6-phosphate and glucose-1-phosphate were lower in leaves and higher in roots than in CK. The relative content of glucose-1-phosphate and D-fructose 6-phosphate in stems was higher than CK in the 4m pruning treatment, but the relative content of D-glucose 1,6-bisphosphate in stems was lower than CK. Heat map analysis revealed some genes, including 12 genes encoding beta-glucosidase (3.2.1.21), 14 genes encoding glucan endo-1,3-beta-D-glucosidase (3.2.1.39), three gene encoding hexokinase (2.7.1.1), three genes encoding fructokinase (2.7.1.4), eight genes encoding endoglucanase (3.2.1.4), one gene encoding maltase-glucoamylase (3.2.1.20), one gene encoding sucrose synthase (2.4.1.13), one gene encoding sucrose-phosphate synthase (2.4.1.14), one gene encoding phosphoglucomutase (5.4.2.2), and one gene encoding beta-fructofuranosidase (3.2.1.26). We found that one gene encoding 2.4.1.14 and one encoding 5.4.2.2 was highly expressed in stems among all pruning intensities. One gene encoding 3.2.1.20 and one encoding 2.4.1.13 was highly expressed in leaves among all pruning intensities. Most genes encoding 3.2.1.39 and 3.2.1.4 were expressed in stems at all pruning intensities. Furthermore, the genes encoding 3.2.1.21, 2.7.1.1, 2.7.1.4, and 3.2.1.26 showed both up/downregulated genes under different pruning intensities in all parts. These results suggest that the identified DEGs and DEMs play key roles in starch and sucrose metabolism pathways.

### 2.6. Correlation Analysis of the Genes and Metabolite

**Figure 6:**
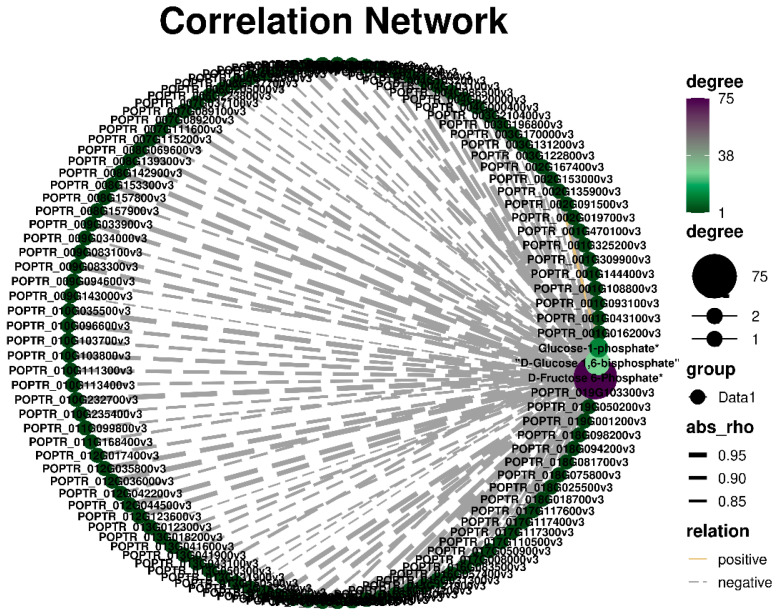
Correlation analysis between structural genes and differential metabolites in starch and sucrose metabolism pathways.

**Figure 7:**
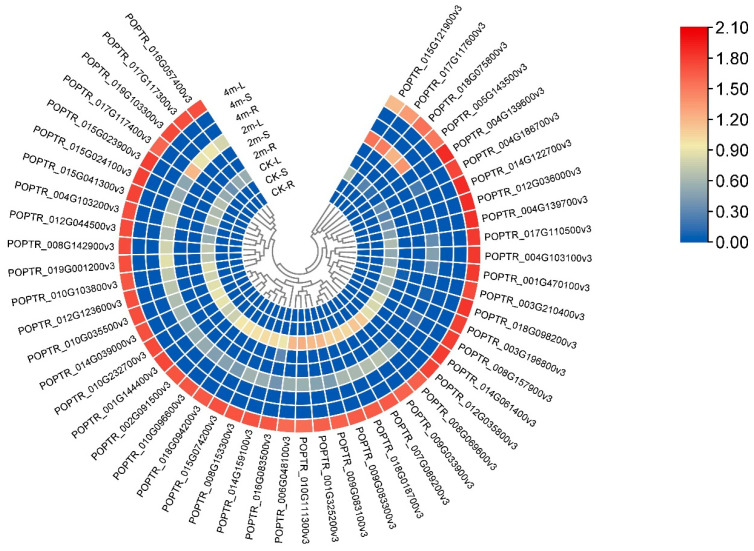
Expression analysis of structural genes associated with differential metabolites in starch and sucrose metabolism pathways.

In this study, we identified genes related to different metabolites in the starch and sucrose metabolic pathways. We analyzed the correlation between metabolites and related genes (Figure 6). The correlation coefficients were greater than 0.8. Most genes were negatively correlated with metabolites, and only two genes (*POPTR_001G108800v3* and *POPTR_002G135900v3*) were positively correlated with glucose-1-phosphate. Based on the correlation analysis results, we conducted a clustering heat map analysis of the genes with a coefficient of 0.9 or more. The results showed that the expression of all genes in the leaves was the highest under 4m pruning intensity (Figure 7). The expression level of 2m pruning intensity was lowest in the stem and root. The correlation and heat map results showed that the lower the gene expression, the higher the content of encoding metabolites. We found three simultaneous genes, namely *POPTR_016G057400v3*, *POPTR_015G041300v3*, and *POPTR_006G048100v3*, in the correlation and pathway analyses. The three genes are involved in glucose synthesis.

### 2.7. Interaction Network of Structural Genes and TFs

**Figure 8:**
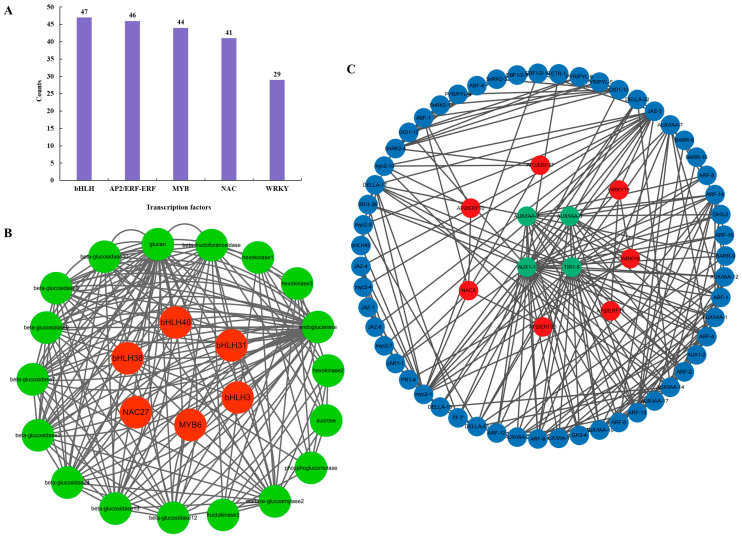
(**A**) Top five TF families based on transcriptome analysis. (**B**) Interaction networks of structural genes and TFs related to starch and sucrose metabolic pathways. (**C**) Interaction networks of structural genes and TFs related to plant hormone signal transduction.

TFs can specifically regulate the expression of structural genes, thereby regulating the biosynthesis of starch and sucrose and plant hormones. In this study, we used transcriptome analysis to identify the top five TF families (bHLH, AP2/ERF-ERF, MYB, NAC, and WRKY). We conducted network analysis of interaction between structural genes and the first five transcription factors in the two pathways, respectively, to obtain the regulatory relationship between structural genes and TFs (Figure 8). In starch and sucrose metabolism pathways, the results showed that bHLH members *POPTR_009G081400v3*, *POPTR_015G104200v3*, *POPTR_001G287200v3*, *POPTR_005G121900v3*, NAC member *POPTR_017G086200v3*, and MYB member *POPTR_006G097300v3* have complex regulatory relationships with some structural genes, and the proportion of bHLH was high. In plant hormone signal transduction, the results showed that AP2/ERF-ERF member *POPTR_001G110800v3*, *POPTR_004G141200v3*, *POPTR_015G136400v3*, *POPTR_001G110700v3*, WRKY member *POPTR_016G137900v3*, *POPTR_018G019700v3*, and NAC member *POPTR_011G123300v3* have complex regulatory relationships with some structural genes, and the proportion of AP2/ERF-ERF was high.

### 2.8. qRT-PCR

**Figure 9:**
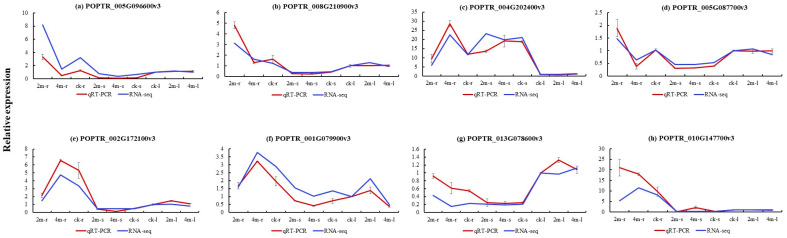
Relative expression of structural genes in the root, stem, and leaf samples. Relative expression of (**a**) *POPTR_005G096600v3*, (**b**) *POPTR_008G210900v3*, (**c**) *POPTR_004G202400v3*, (**d**) *POPTR_005G087700v3*, (**e**) *POPTR_002G172100v3*, (**f**) *POPTR_001G079900v3*, (**g**) *POPTR_013G078600v3*, and (**h**) *POPTR_010G147700v3* in different parts.

**Figure 10:**
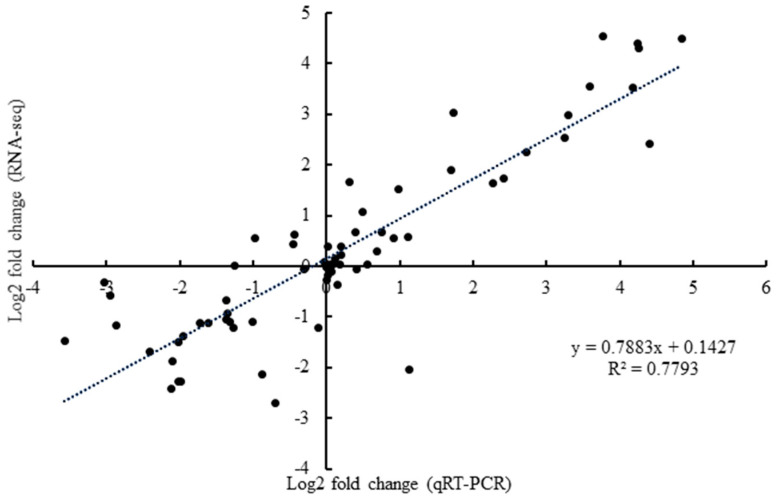
Correlation between RNA-seq and qRT-PCR in root, stem, and leaf samples.

We conducted qRT-PCR to verify the accuracy and reliability of transcriptome data (Figure 9 and Figure 10). Eight representative DEGs (*POPTR_005G096600v3*, *POPTR_008G210900v3*, *POPTR_004G202400v3*, *POPTR_005G087700v3*, *POPTR_002G172100v3*, *POPTR_001G079900v3*, *POPTR_013G078600v3*, and *POPTR_010G147700v3*) were selected for qRT-PCR. These results indicated that the RNA-seq data closely matched the expression patterns of the DEGs. This suggests that our RNA-seq data can effectively identify key genes involved in the starch and sucrose pathways.

## 3. Discussion

### 3.1. Changes in Plant Hormones and Their Regulatory Function Under Different Pruning Intensities

Plant growth is closely related to temperature, light, density, nutrient content, and plant hormones. Pruning is an important measure for plantation management and cultivation. The nutrient substance distribution among the trunk, branches, and leaves can be adjusted reasonably by artificially pruning dead branches and dense branches in the poplar lower part to make the trunk straight and promote forest growth. Plant hormones are signaling molecules that play crucial roles in numerous intricate signal transduction pathways, including those involving ABA, MAPK, and protein phosphorylation. These hormones also interact to coordinate and regulate various aspects of plant growth, development, and responses to biotic and abiotic stresses. In this study, “plant hormone signal transduction” was a significantly enriched pathway, and the contents of auxin, GA, and brassinolide significantly changed in different parts under different pruning treatments, indicating that they played a key role in growth and development after pruning. Auxin is mainly distributed in the cambium region, while GA is primarily distributed in the developing xylem [15,16]. Different hormones play different roles. Brassinolide is an important phytohormone that regulates plant growth and development and the interaction between plants and the environment. It can enhance crop yield, quality, and stress resistance. Simultaneously, several hormones may interact with one another to coordinate plant vascular development. In GA biosynthesis and signal transduction pathways, the TF structure gene is upregulated in the stem, especially in 2m-S, promoting stem growth. Auxin has long been regarded as the most important hormone affecting plant growth and development, forming a cumulative peak in cambium and playing an important role in maintenance, division, and differentiation [15,17]. AUX/IAA, ARF, AUXI, and TIR1 structural genes were highly expressed in AUXI biosynthesis and signal transduction pathways, and AUXI expression was higher than CK under pruning treatment. Brassinolide has been shown to control cell division and expansion [18]. In BR signal transduction, phosphorylation of protein kinases and phosphorylation and dephosphorylation of TFs are the intrinsic biochemical mechanisms that regulate plant trait development and environmental adaptability. The protein kinase Brassinocorticosteroid-insensitive 1 (BRI1), whose initial signal is extracellular transduction into intracellular BRI1-associated receptor kinase 1 (BAK1), plays an important role in plant growth and immune balance. Additionally, TFs brassinazole-resistant 1 (*BZR1*) and *BZR2*, regulating gene expression of different traits downstream of the BRs signal, are key genes in the BRs signaling pathway. *BZR1/2* binds to different trait genes and is involved in regulating stomatal development [19], seed morphology [20], and improving tomato quality [21]. *BZR1* also interacted with proteins such as phytochrome interacting factors 4 (*PIF4*), regulating plant cell growth, photomorphogenesis, and plant type [22]. The structural genes of the brassinolactone signal transduction pathway were differentially expressed in different parts of all pruning treatments, suggesting that they may be widely involved in transcriptional regulation and provide key molecular signals for downstream reactions. In summary, these results indicate that Auxin, GA, and brassinolide play an important role in the growth and development of poplar.

### 3.2. Changes of Metabolites in Starch and Sucrose Metabolism Pathway and Their Regulatory Functions Under Different Pruning Intensities

Starch is a storage form of photosynthetic products, and sucrose is a precursor of starch synthesis. Green plants produce carbohydrates by photosynthesis during the day using carbon dioxide in the atmosphere, water, and a series of enzymes. The resulting assimilation products were divided into sucrose and starch, and the latter was stored to meet the plant’s night respiration consumption needs. However, synthetic sucrose is mainly active during the day [23]. Sucrose formed by plant cells during photosynthesis is eventually decomposed and used to form a hexose. The synthesized sucrose has two destinations for maintaining plant metabolism. Part of it is transported to the vacuole by sucrose transporters on the cell membrane and then decomposed into fructose and glucose by invertase (Inv). The energy necessary for the normal development of various tissues and organs of the plant is mainly supplied by sucrose, whereas the others are transferred to these tissues and organs. In this study, the expression levels of D-fructose-6-phosphate, glucose-1-phosphate, and glucose-1, 6-diphosphate in stems were higher in the treatment group than in the CK group, and the expression levels of glucose-1-phosphate and glucose-1, 6-diphosphate were the highest in 2m-S. After pruning, the relative contents of three different metabolites in stems were significantly increased, and the relative contents in stems of the 2m group were higher than CK. In the 4m group, the relative content of glucose-1,6-diphosphate in stems was lower than in CK, while D-fructose-6-phosphate and glucose-1-phosphate were higher than in CK. After pruning, the relative contents of D-fructose-6-phosphate and glucose-1-phosphate in leaves were lower than CK but higher than CK in roots. The results indicated that the nutrient distribution in poplar changed after pruning, and the DBH in the pruning group was higher than that in the CK group. This may be due to the fact that the lateral branches in the lower layer of the canopy cannot effectively conduct photosynthesis and synthesize nutrients necessary for tree growth in this planting density. Instead, the nutrients from the trunk were consumed. However, the consumption of trunk nutrients is reduced after pruning, and the nutrients available for the trunk’s growth increase compared to the CK group, thereby promoting the growth of the tree. Consequently, it shows that starch and sucrose play a key role in the growth and development of plants, similar to Coleman’s study on wood [24], indicating that appropriate pruning can adjust the substance distribution of individual plants and promote plant growth and wood yield [1,25,26].

### 3.3. TFs Involved in Regulating Starch and Sucrose Synthesis

Transcription factors (TFs) are important regulatory factors in plant development. In this study, the top five TFs were analyzed based on transcriptome analysis, namely bHLH, AP2/ERF-ERF, MYB, NAC, and WRKY. The interaction network analysis of structural genes and TFs of the two pathways was carried out, respectively. The result shows that the synergistic regulation of bHLH, MYB, NAC, and other TFs regulate starch and sucrose biosynthesis. TFs such as AP2/ERF-ERF, NAC, and WRKY regulate hormone synthesis by regulating structural genes related to the plant hormone signal transduction pathway, especially in auxin.

In previous studies, MdbHLH3 in *Malus domestica* was directly bound to the promoter of apple cytoplasmic malate dehydrogenase (*MdcyMDH*). It activates its transcriptional expression, thereby promoting malic acid accumulation in apples. Additionally, MdbHLH3 overexpression increased the photosynthetic capacity and carbohydrates in apple leaves. It enhanced the carbohydrate accumulation in fruits by regulating its distribution between leaf and fruit, confirming that starch and sucrose biosynthesis was significantly regulated by bHLH TFs [27]. Similarly, MYB transcription factors (*TSF1-4*) have been verified to activate glycoprotein gene expression in potatoes [28]. Additionally, NAC transcription factors are a large family of plant-specific TFs involved in almost the entire process of plant growth and development. Relevant studies have shown that NAC family TFs significantly affect BAK1’s participation in regulating plant growth and development under strong light [29]. These results suggest that starch and sucrose synthesis pathways are regulated by some key TFs, namely bHLH, MYB, and NAC, of which bHLH plays a major role, similar to *Malus domestica* [27].

AP2/ERF-ERF transcription factors are involved in plant growth and development. Previous studies have shown that AP2/ERF-ERF transcription factors (*CRL5* and *OsERF3*) can participate in crown root formation [30] and elongation [31] in *Oryza sativa* L.. According to Neogy et al., *OsAP2/ERF-40* can promote the development of adventitious root in *O. sativa*. [32]. Trupiano found that *PtaERF003* can positively regulate adventitious root and lateral root proliferation in poplar [33]. In chrysanthemum studies, *CmERF053* was found to regulate plant branches and lateral roots [34]. In *Arabidopsis thaliana* (L.) Heynh., it was found that *BOLITA* can adjust leaf size [35]. In tomato, it was found that *eERF2* promoted fruit ripening, and *PpIAA1* and *PpERF4* regulated peach fruit ripening by integrating auxin and ethylene signals to form a positive feedback loop [36]. In summary, AP2/ERF-ERF transcription factors play a key role in plant hormone synthesis and plant growth and development, which may regulate hormone synthesis in plants by regulating related genes, especially in regulating auxin.

## 4. Materials and Methods

### 4.1. Plant Materials and Site

In this study, the sample material was from 11-year-old Populus euramericana “N3016” × *P. suriensis* from the Xinjiang Experimental Forest Farm in Nehe City, Qiqihar City, Heilongjiang Province (124°22′30″–124°30′00″ E, 47°58′30″–48°05′00″ N). The annual mean temperature of the site is 0.7℃, the maximum temperature is 36.5 °C, and the minimum temperature is −39 °C. The annual precipitation at the site is 430 mm, with a frost-free period of 126–130 days and a total sunshine duration of 2715 h throughout the year. Influenced by the continental monsoon climate, the area experiences little rainfall and windy conditions in early spring, with precipitation concentrated in July and August during summer. Early frosts are common in autumn, and winters are severely cold. The soil types are mainly composed of alluvial sandy soil and chernozem, with a granular soil structure.

**Figure 11:**
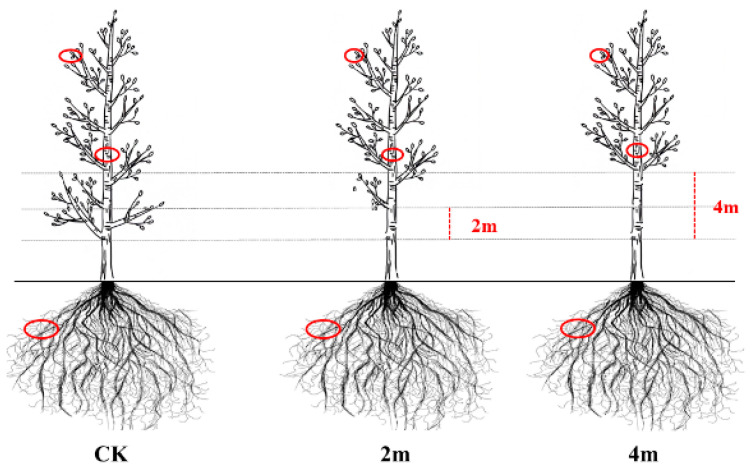
The different pruning treatment of 11-year-old poplar trees. (CK, no pruning. 2m, the height under the branches after pruning, accounts for 35% of the tree height. 4m, the height under the branches after pruning, accounts for 45% of the tree height).

In March 2022, pruning treatment at three intensities was conducted in the stand with a planting density of 2 × 3 × 6 m, respectively. The pruning intensities are 2m, 4m, and CK, respectively. Among them, the pruning intensity of 2m refers to the condition where the height under the branches after pruning accounts for 35% of the tree height. The pruning intensity of 4 m refers to the condition where the height under the branches after pruning accounts for 45% of the tree height, and CK is not pruning. The experimental design is shown in Figure 11.

The diameter at breast height (DBH) data were measured before and after pruning treatment. In early August, the stems of unpruned areas, leaves, and roots of the same height and direction under all treatments were collected from 9:00 a.m. to 11:00 a.m. when the weather was clear. The samples were labeled and stored in liquid nitrogen for subsequent experiments.

### 4.2. Detection and Analysis of Primary Metabolites

This study included primary metabolome assays and transcriptome sequencing and was commissioned by Wuhan Metviare Biotechnology Co., Ltd. (Wuhan, China).

Samples were extracted using standard extraction methods for UPLC-MS/MS analysis. The mass spectrum data were processed using Analyst 1.6.3. Quality control and CV value calculations were conducted to obtain high-quality data. The data were evaluated and statistically analyzed.

According to the results of metabolite determination, the relative content of differential metabolites in the metabolic pathway was extracted, and the changes of differential metabolites in different pruning treatments, stems, and leaves were analyzed by line graph.

### 4.3. Library Construction and RNA Sequencing

The tissues were subjected to the hexadecyl trimethyl ammonium bromide (CTAB) extraction method to isolate RNA. To obtain mRNA, ribosomal RNA was extracted from the total RNA and subsequently fragmented using a fragmentation buffer. The short-fragment RNA served as a template for synthesizing the first cDNA strand, utilizing random hexamers in the process. Double-stranded cDNA was synthesized by adding the buffer, dNTPs, and DNA polymerase I to the reaction mixture. AMpure XP beads were employed to purify the double-stranded cDNA, which was then subjected to end-repair, the addition of an A-tail, and ligation of sequencing adapters. Using AMpure XP beads, fragment sizes were isolated, and the final cDNA library was obtained through PCR enrichment.

### 4.4. Library Quality Inspection and RNA Sequencing

After constructing the library, the insert size of the library was determined using the Agilent 2100 Bioanalyzer (Agilent, Santa Clara, CA, USA). The effective concentration of the library was accurately quantified using the quantitative polymerase chain reaction (qPCR), and the effective concentration of the library was > 2 nm. Qualified samples were sequenced using Illumina (San Diego, CA, USA).

### 4.5. Sequencing Data Quality Control and Data Analysis

Filtering the original data is essential to guarantee the quality and reliability of the subsequent data analysis. This encompassed discarding adapter reads, paired reads with an N content surpassing 10% of the read base number in any sequenced read, as well as reads of low quality (those with Qphred ≤ 20 base number, accounting for more than 50% of the entire read length). The genome of *Populus trichocarpa* Torr. & Gray obtained from the National Center for Biotechnology Information (NCBI) was selected as a reference.

HISAT2 software (Version 2.0.5) was employed to build the reference genome index, and the paired-end clean reads were aligned to this reference genome using the same version of HISAT2. According to the gene expression level in different sample groups, differential expression analysis, functional annotation, and functional enrichment of differential expression genes were performed. HTseq software (version 0.6.1) was utilized to quantify the number of genes at varying expression levels and to ascertain the expression levels of individual genes. Differentially expressed genes (DEGs) were determined using DESeq2 (version 1.22.1), and the correction values ranged between a false discovery rate (FDR) <0.05 and |log2 fold-change| ≥ 1. Differentially expressed genes were analyzed for gene ontology (GO) (version 2022.0915) enrichment using the Goseq R (version 3.24.3) package. Kyoto Encyclopedia of Genes and Genomes (KEGG) (version 2022.10) pathway enrichment analysis of differentially expressed genes was performed using KOBAS 2.0 software.

### 4.6. Quantitative Real-Time PCR (qRT-PCR) Verification

To validate the RNA-seq data, eight genes were chosen for qRT-PCR, with each gene being subjected to three technical replicates. An online tool was utilized to design primers for all the candidate genes (https://sg.idtdna.com/scitools/Applications/RealTimePCR/default.aspx, accessed on 15 November 2023). ACTIN, known for its stable expression and resistance to RNA degradation, was chosen as the internal reference gene. Total RNA was isolated from the samples, and subsequently, cDNA was synthesized using a cDNA Synthesis Kit (Takara, Kyoto, Japan). The qRT-PCR reactions were then performed with 2× TB Green PCR Master Mix (Takara, Kyoto, Japan) on a 7500 Fast Real-time PCR System. Each reaction comprised a 20 µL mixture, consisting of 10 µL of 2x SYBR Premix Ex Taq, 2 µL of cDNA template, 6 µL of ddH2O, 0.8 µL of upstream and downstream primers, and 0.4 µL of ROX reference dye. The qRT-PCR procedure was as follows: 95 °C for 30 s, followed by 45 cycles at 94 °C for 5 s, 60 °C for 35 s, 95 °C for 15 s, 60 °C for 1 min, and 95 °C for 15 s. The relative expression levels of all DEGs were calculated using the 2^−∆∆Ct^ method [37].

### 4.7. Statistical and Visualization Analyses

Statistical Package for Social Sciences (SPSS, version 26.0) and Excel 2019 were utilized for data analysis. Additionally, TBtools 2.140 software was employed for visualization analysis, generating heat maps and Venn plots.

## 5. Conclusions

This study measured and analyzed the DBH of the two pruning treatments and the CK group. This study found that the mean DBH of the treatment group was higher than that of the CK group, and DBH growth was better under a 2m pruning intensity. We used LC-MS/MS to detect the content of primary metabolites in poplar roots, stems, and leaves under different pruning treatments and performed transcriptomic analysis. After pruning, the biosynthesis and expression patterns of three key plant hormones, GA, IAA, and BR, in the signal transduction pathway were evaluated. A total of 7316 DEGs and three DEMs were screened using transcriptomic and metabolomic methods. Enrichment analysis of DEG and DEM showed that starch and sucrose biosynthesis was common in the comparison group. According to the correlation and pathway analysis results, three genes were involved in glucose synthesis. We proposed the accumulation patterns of key metabolites, such as D-fructose 6-phosphate, D-glucose 1,6-bisphosphate, and glucose-1-phosphate. The expression levels of the three metabolites in the stems were higher in the treatment group than in the CK group. The expression levels of glucose-1-phosphate and glucose-1, 6-diphosphate were highest at 2m-S. We further predicted that beta-glucosidase, endoglucanase, hexokinase, glucan endo-1, 3-beta-D-glucosidase, beta-fructofuranosidase, fructokinase, maltase-glucoamylase, phosphoglucomutase, and sucrose are involved in starch and sucrose synthesis. We also identified bHLH, MYB, and NAC TFs involved in the starch and sucrose biosynthesis pathways. Four bHLH TFs, one MYB TF, and one NAC TF were obtained and inter-regulated with structural genes related to starch and sucrose biosynthesis pathways. These results provide valuable resources for studying molecular regulation mechanisms and synthetic biology of starch and sucrose. In conclusion, the distribution of nutrients among the trunk, branches, and leaves can be adjusted by pruning the stand at an appropriate intensity to promote forest growth.

## Data Availability

The raw data have been uploaded to the NCBI SRA database (BioProject ID: PRJNA1115892).

## Supplementary Materials

The following supporting information can be downloaded at: https://www.mdpi.com/article/10.3390/plants13233391/s1, Table S1: The summary of sequencing data.

## References

[1] Xiao X.X. (2005). Effect of stem pruning on growth and knot free timber production of *Fokienia hodginsi* plantation. For. Res..

[2] Guo M.H. (2003). The effect of silvicultural measures on radial growth properties of *Pinus Koraiensis* plantations. Sci. Silvae Sin..

[3] Chen Z.Z., Zhang Y.Q., Bin W.U., Zhi-Pei L.I., Geng X.G., Feng J.Y., Tian S.Y., Lei N. (2012). Effect of shoot pruning on light intensity and wheat yield near farmland shelterbelt. J. Triticeae Crops.

[4] Dong X., Chi Y.C., Hao Y.G., Chen H.L., Yang Z.Y., Liu N., Zhao Y.M., Huang Y.R. (2021). Effect of pruning years on photosynthetic characteristic and specific leaf area of *Ammopiptanthus mongolicus*. J. Cent. South Univ. For. Technol..

[5] Wang M.F. (2013). Analyse the Genetic Relationship of the Native Populus in Sichuan by ISSR and ITS Sequences. Master’s Thesis.

[6] Guo P., Jin H., Yin W.L., Xia X.L., Jiang G.F. (2012). The cloning and expression of WUE-related gene (PdEPF1) in *Populus deltoides×Populus nigra*. Acta Ecol. Sin..

[7] Zhang Q.W. (1999). Poplar excellent pulp and ecological shelterbelt new variety European American Yang No. 107. For. Res..

[8] Ning K., Ding C., Huang Q., Zhang W., Su X. (2019). Transcriptome profiling revealed diverse gene expression patterns in poplar (*Populus × euramericana*) under different planting densities. PLoS ONE.

[9] Zhang H.Y. (2021). Determination of Cold Resistance and Fingerprint Construction of Poplar Clones. Master’s Thesis.

[10] Wang J. (2006). Prediction and Control of Main Leaf Eating Insect and Mulberry Longicorn on Poplar in Binzhou. Master’s Thesis.

[11] Liu W., Man S.J., Su X.H., Peng R.S., Wu J.J. (2022). Analysis and identification of section aigeiros germplasm based on SSR molecular markers. J. Southwest For. Univ. (Nat. Sci.).

[12] Zhao X.Q. (2023). Phenptypic Identification of SpsTACs Gene Overexpression and Homology Knockout in Poplars. Master’s Thesis.

[13] Wang R., Wang G.B., Xu J., Xu X. (2021). Effects of litterfalls and earthworms on distribution of soil aggregates and carbon and nitrogen content in poplar plantations. J. Nanjing For. Univ. (Nat. Sci. Ed.).

[14] Qiao H.H., Li H.B., Sun Z.M., Ke Q.B., Wang S.Y., Guo S.Z., Deng X.P. (2019). Physiological mechanisms of transgenic poplar with enhancing tolerance to drought stress by expression IbLEA14 gene. Mol. Plant Breed..

[15] Immanen J., Nieminen K., Smolander O.P., Kojima M., Serra J.A., Koskinen P., Zhang J., Elo A., Mahonen A.P., Street N. (2016). Cytokinin and auxin display distinct but interconnected distribution and signaling profiles to stimulate cambial activity. Curr. Biol..

[16] Israelsson M., Sundberg B., Moritz T. (2005). Tissue-specific localization of gibberellins and expression of gibberellin-biosynthetic and signaling genes in wood-forming tissues in aspen. Plant J..

[17] Fischer U., Kucukoglu M., Helariutta Y., Bhalerao R.P. (2019). The dynamics of cambial stem cell activity. Annu. Rev. Plant Biol..

[18] Ohtaka K., Yoshida A., Kakei Y., Fukui K., Kojima M., Takebayashi Y., Yano K., Imanishi S., Sakakibara H. (2020). Difference between day and night temperatures affects stem elongation in tomato (*Solanum lycopersicum*) seedlings via regulation of gibberellin and auxin synthesis. Front. Plant Sci..

[19] Khan M., Rozhon W., Bigeard J., Pflieger D., Husar S., Pitzschke A., Teige M., Jonak C., Hirt H., Poppenberger B. (2013). Brassinosteroid-regulated GSK3/Shaggy-like kinases phosphorylate mitogen-activated protein (MAP) kinase kinases, which control stomata development in Arabidopsis thaliana. J. Biol. Chem..

[20] Jiang W.B., Huang H.Y., Hu Y.W., Zhu S.W., Lin W.W.H. (2013). Brassinosteroid regulates seed size and shape in Arabidopsis. Plant Physiol..

[21] Liu L.H., Jia C.G., Zhang M., Chen D.L., Chen S.X., Guo R.F., Guo D.P., Wang Q.M. (2014). Ectopic expression of a BZR1-1D transcription factor in brassinosteroid signalling enhances carotenoid accumulation and fruit quality attributes in tomato. Plant Biotechnol. J..

[22] Casal J.J. (2013). Photoreceptor signaling networks in plant responses to shade. Annu. Rev. Plant Biol..

[23] Smith A.M., Stitt M. (2007). Coordination of carbon supply and plant growth. Plant Cell Environ..

[24] Coleman H.D., Yan J., Mansfield S.D. (2009). Sucrose synthase affects carbon partitioning to increase cellulose production and altered cell wall ultrastructure. Proc. Natl. Acad. Sci. USA.

[25] Newton M., Lachenbruch B., Robbins J.M., Cole E.C. (2012). Branch diameter and longevity linked to plantation spacing and rectangularity in young Douglas-fir. For. Ecol. Manag..

[26] Zhang T., Zhu Y.J., Dong X.B. (2017). Effects of thinning and pruning on the growth and canopy of larch forest. J. Northeast. For. Univ..

[27] Yu J.Q., Gu K.D., Sun C.H., Zhang Q., Hao Y. (2021). The apple bHLH transcription factor MdbHLH3 functions in determining the fruit carbohydrates and malate. Plant Biotechnol. J..

[28] Choi H., Baek S.Y., Kim S.Y. (2017). MYB class transcription factors bind to the tuber-specific and sucrose-response element of a class-I patatin promoter. Plant Biotechnol. Rep..

[29] Zhang S. (2020). Molecular Mechanisms of BAK1 and SINAC4 Regulating Plant Growth and Development. Ph.D. Thesis.

[30] Kitomi Y., Ito H., Hobo T., Aya K., Kitano H., Inukai Y. (2011). The auxin responsive AP2/ERF transcription factor CROWN ROOTLESS5 is involved in crown root initiation in rice through the induction of OsRR1, a type-A response regulator of cytokinin signaling. Plant J..

[31] Zhao Y., Cheng S.F., Song Y.L., Huang Y.L., Zhou S.L., Liu X.Y., Zhou D.X. (2015). The interaction between rice ERF3 and WOX11 promotes crown root development by regulating gene expression involved in cytokinin signaling. Plant Cell.

[32] Neogy A., Garg T., Kumar A., Dwivedi A.K., Singh H., Sing U., Singh Z., Prasad K., Jain M., Yadav S.R. (2019). Genome-wide transcript profiling reveals an auxin-responsive transcription factor, OsAP2/ERF-40, promoting rice adventitious root development. Plant Cell Physiol..

[33] Trupiano D., Yordanov Y., Regan S., Meilan R., Tschaplinski T., Scippa G.S., Busov V. (2013). Identification, characterization of an AP2/ERF transcription factor that promotes adventitious, lateral root formation in *Populus*. Planta.

[34] Nie J., Wen C., Xi L., Lv S., Zhao Q., Kou Y., Ma N., Zhao L., Zhou X. (2018). The AP2/ERF transcription factor CmERF053 of chrysanthemum positively regulates shoot branching, lateral root, and drought tolerance. Plant Cell Rep..

[35] Marsch-Martinez N., Greco R., Becker J.D., Dixit S., Bergervoet J.H.W., Karaba A., Folter S., Pereia A. (2006). BOLITA, an Arabidopsis AP2/ERF-like transcription factor that affects cell expansion and proliferation/differentiation pathways. Plant Mol. Biol..

[36] Wang X.B., Pan L., Wang Y., Meng J., Deng L., Niu L., Liu H., Ding Y.F., Yao J.L., Nieuwenhuizen N.J. (2021). PpIAA1 and PpERF4 form a positive feedback loop to regulate peach fruit ripening by integrating auxin and ethylene signals. Plant Sci..

[37] Livak K.J., Schmittgen T.D. (2001). Analysis of relative gene expression data using real-time quantitative PCR and the 2^−ΔΔCT^ method. Methods.

